# Understanding the mechanism and regioselectivity of the copper(i) catalyzed [3 + 2] cycloaddition reaction between azide and alkyne: a systematic DFT study†‡

**DOI:** 10.1039/c7ra10653j

**Published:** 2018-02-16

**Authors:** Hicham Ben El Ayouchia, Lahoucine Bahsis, Hafid Anane, Luis R. Domingo, Salah-Eddine Stiriba

**Affiliations:** Laboratoire de Chimie Analytique et Moléculaire, LCAM, Faculté Polydisciplinaire de Safi, Université Cadi Ayyad Safi 46030 Morocco ananehafid@gmail.com +212-5-24669517; Departamento de Química Orgánica, Universidad de Valencia Avda. Dr Moliner 50, 46100 Burjassot Valencia Spain; Instituto de Ciencia Molecular/ ICMol, Universidad de Valencia C/Catedrático José Beltrán No. 2, 46980 Paterna Valencia Spain stiriba@uv.es +34-96-354-4445

## Abstract

The copper(i) catalyzed azide–alkyne [3 + 2] cycloaddition (32CA) reaction and its uncatalyzed version have been studied for systematic understanding of this relevant organic transformation, using DFT calculations at the B3LYP/6-31G(d) (LANL2DZ for Cu) computational levels. In the absence of a copper(i) catalyst, two regioisomeric reaction paths were studied, indicating that the 32CA reaction takes place through an asynchronous one-step mechanism with a very low polar character. The two reactive channels leading to 1,4- and 1,5-regisomer present similar high activation energies of 18.84 and 18.51 kcal mol^−1^, respectively. The coordination of copper(i) to alkyne produces relevant changes in this 32CA reaction. Analysis of the global and local electrophilicity/nucleophilicity allows explaining correctly the behaviors of the copper(i) catalyzed cycloaddition. Coordination of the copper to alkyne changes the mechanism from a non-polar one-step mechanism to a polar stepwise one, as a consequence of the high nucleophilic character of the dinuclear Cu(i)-acetylide complex. Parr and Fukui functions and Dual Descriptor correctly explain the observed regioselectivity by means of the most favorable two-center interaction that takes place along the 1,4 reaction path.

## Abstract 2

Analysis of the global and local electrophilicity/nucleophilicity indices allows correct explanation of the behaviors of the copper(i) catalyzed 32CA reaction.

## Graphical abstract

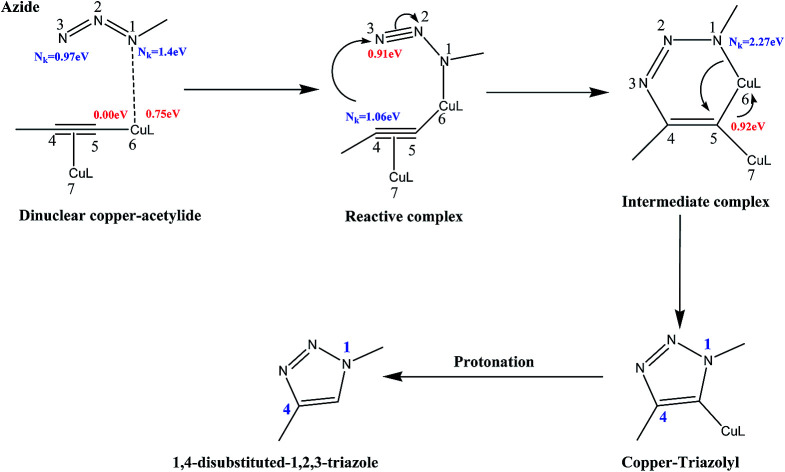

## Introduction

**Scheme 1:**
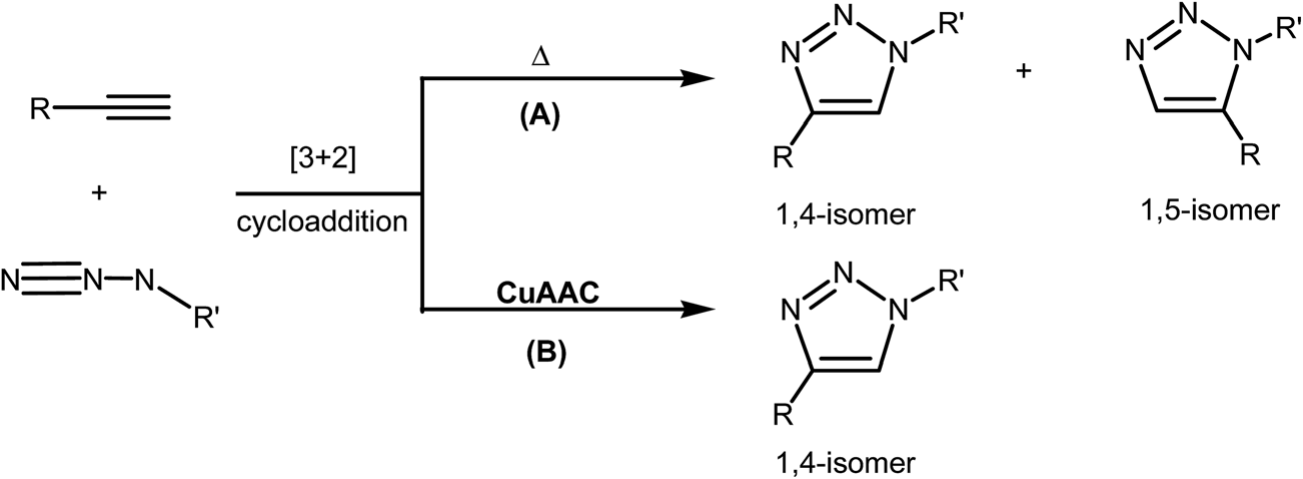
Uncatalyzed thermal (A) and copper(i) catalyzed (B) azide–alkyne 32CA reaction.

The [3 + 2] cycloaddition (32CA) reactions of a three-atom-component (TAC) such as organoazides with alkynes or alkenes have been known for more than a century.^1^ However, the rational discovery of this type of cycloaddition reaction was only elucidated by R. Huisgen in 1960, who coined the topological term 1,3-dipolar cycloaddition reaction.^2^ This reaction was recently brought back into the front of the family of elegant synthetic methodologies by Meldal and Sharpless who devised a Cu(i)-catalyzed azide–alkyne cycloaddition (CuAAC) reaction.^3^ Such synthetic approach fits well within the concept of “*click chemistry*” that aims to produce substances by clicking selective components. In fact, CuAAC proceeds at room temperature, leading to the regiospecific formation of heterocyclic 1,4-disubstituted 1,2,3-triazol in excellent yield as illustrated in Scheme 1.^4^

**Fig. 1:**
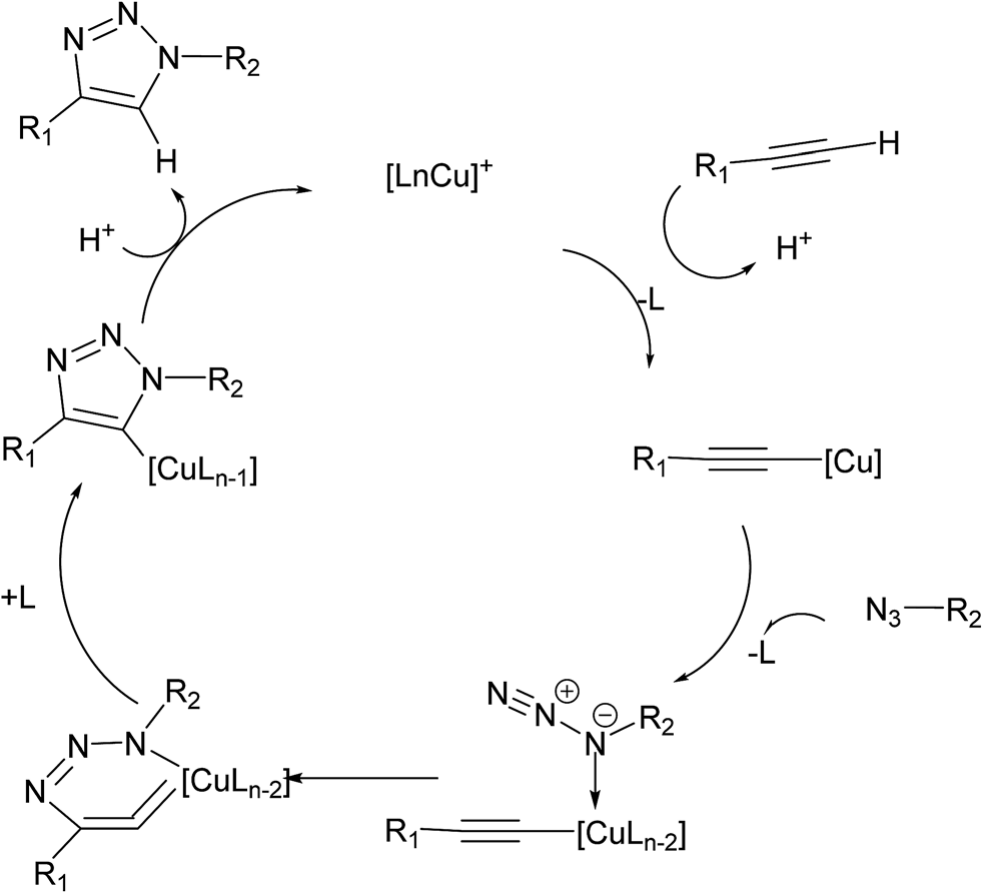
Early CuAAC mechanism proposed by Sharpless and Fokin.

In their seminal report on the use of Cu(i)-catalyzed 32CA reaction of azides with terminal alkynes, Sharpless and Fokin proposed an early mechanism (Fig. 1) that has served as a surprisingly good starting point for further posterior mechanistic investigations.^3^

They later reported the results of an extensive DFT^5^ study in 2005, finding that an activation barrier of 23.7 kcal mol^−1^ rules out a one-step mechanism from the neutral Cu(i)-acetylide. Their calculations also predicted the activation barrier for the formation of a six-membered copper(iii) metallacycle to be 14.9 kcal mol^−1^ compared to a barrier of 25.7 kcal mol^−1^ for the uncatalyzed cycloaddition, providing then an explanation for the enormous rate acceleration by using copper(i) as catalyst. Interestingly, interaction of copper(i) with the terminal alkyne π-system, namely propyne was found to lower the p*K*_a_ of the terminal proton by almost 10 units facilitating deprotonation, yet reaction from the non-deprotonated Cu(i)-alkyne π-complex was ruled even to be more unfavorable than the uncatalyzed reaction with a barrier of 27.8 kcal mol^−1^.^6^

Based on the formation and spectroscopic identification of copper(i) acetylides by the action of a copper(i) complex with a terminal alkyne and the absence of evidences that point to internal triple bonds undergoing cycloaddition,^7–9^ it is now assumed that formation of copper(i) acetylides is an important activation step being immediately engaged in an efficient sequence affording the formation of 1,4-disubstitued 1,2,3-triazol in a regioselective manner.^7^ While computational studies supported the involvement of Cu(i)-acetylides interacting with organoazides as originally suggested by Sharpless, these complexes were only assumptions until direct evidence was provided by Straub.^8^ In fact, the reaction of an *N*-heterocyclic carbene Cu(i)-acetylide complex with a sterically hindered organoazide led to the formation and isolation of a stable Cu(i)-triazolide complex, which reacted quantitatively with acetic acid to give the expected triazole product within minutes. This isolated intermediate did add more complexity to already conflicting reports on the speciation and nuclearity of copper(i) in the transition state structures (TSs) and intermediates. Kinetic studies have determined the rate law to be first order in azide, between first and second order in alkyne and second order with respect to the copper ions.^10,11^ It was also found that the order of reaction with respect to each reagent to vary relatively and even a negative order has been measured for the concentration of alkyne in certain catalytic systems.^9,12^

**Fig. 2:**
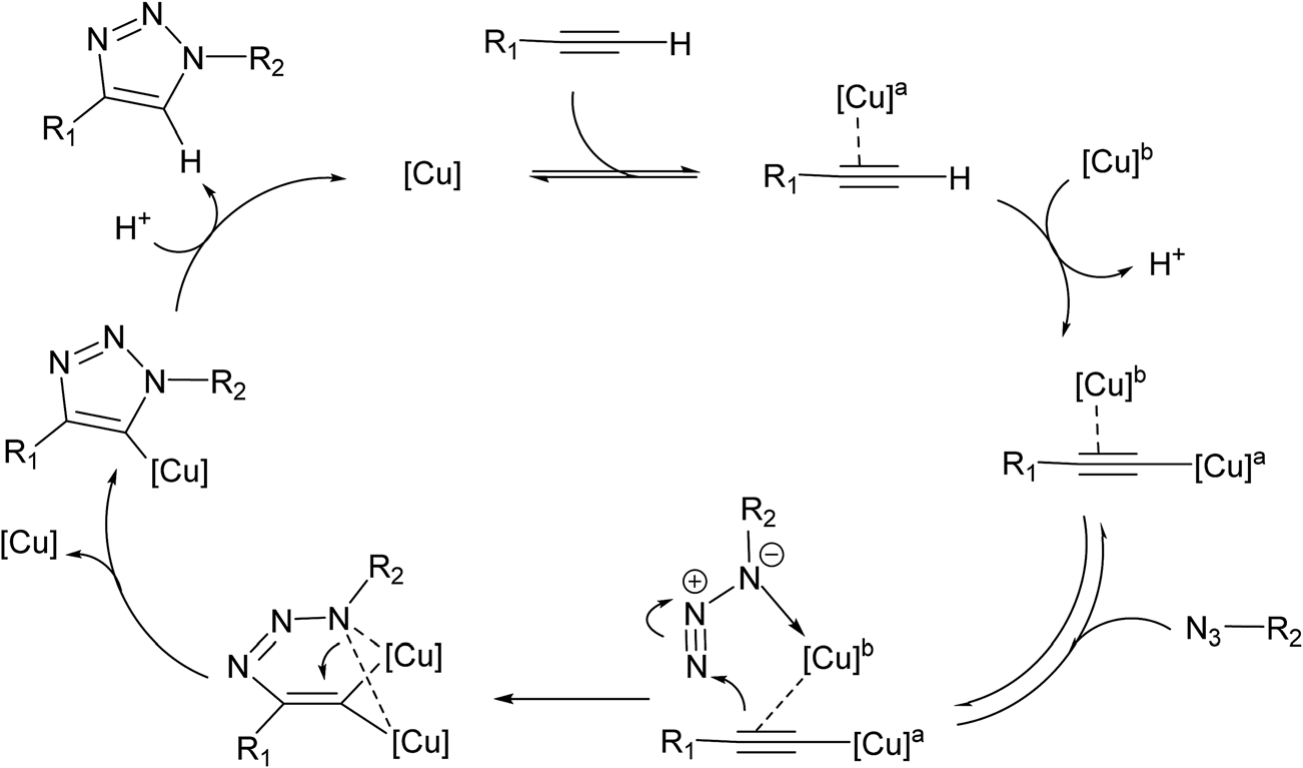
Recent CuAAC mechanism proposed by Fokin.

Recently, Fokin reported that Cu(i)-acetylides only reacted with benzyl azide by adding exogenous Cu(i). In fact, without exogenous Cu(i) added to the reaction mixture, no appreciable conversion of the Cu(i)-acetylide and benzyl azide into triazole products was observed, which supports the hypothesis that a dinuclear species with two copper atoms operate in discrete specialized roles; one as a purely σ-bound ligand, and the other acting solely through weak π-complexation.^13^ Such a postulated π,σ-bis(copper) acetylide intermediate type and a new bis(metallated) triazole complex, viewed as one of the resting states of the catalytic cycle, were elegantly isolated and X-ray structurally characterized by Bertrand, by using the strong properties σ-donating and π-accepting properties of cyclic (alkyl)(amino)carbenes for the isolation of copper metal ions.^14^ Electrospray ionization mass spectrometry (ESI-MS) allowed also the fishing and structural characterization of such a dinuclear copper species as intermediate in CuAAC, using a combination of neutral reactant approach and the ion-tagging strategy.^15^ Previous computational studies have suggested that bridging dicopper(i,iii) μ-alkenylidene fragments are thermodynamically highly stable and show superior reactivity towards organoazides compared to the ring strain in a Cu=C=C intermediate despite the entropically disfavored inclusion of a second copper atom.^16^ The thermodynamic stability of the favored dicopper(i,iii) μ-alkenylidene fragments was attributed to the elimination of high ring strain in the mononuclear six-membered structure. However, isotopic enrichment of triazole products from addition of an isotopically pure ^63^Cu(i) coordination complex to preformed naturally abundant Cu(i)-acetylides (^63^Cu/^65^Cu ratio of 69/31) in the presence of benzyl azide discard the hypothesis of Cu atoms acting in discrete roles and instead suggesting a mechanism in which two chemically equivalent copper atoms work in cooperative manner. Based on these results and previous kinetic studies, Fokin proposed a revisited mechanism whereby a σ-bound Cu(i)-acetylide bearing a π complexed copper atom reacts with an organoazide forming a bridging dicopper μ-acetylide intermediate (Fig. 2).

This mechanistic study represents the most up-to-date picture of the identity of important intermediates and TSs involved in the CuAAC reaction,^14,15^ being supported by the last experimental works that reported the elucidation of the exact structural features of the key dicopper intermediate species responsible for the highest rate acceleration accomplished by Cu(i) catalysis in CuAAC and confirmed by computational studies that had thoroughly proposed such dicnuclear copper intermediates.^17,18^

Recent Molecular Electron Density Theory (MEDT)^19^ studies devoted to 32CA reactions have allowed establishing a very good correlation between the electronic structure of TACs and their reactivity towards ethylene.^20^ Thus, depending on the electronic structure of the TAC, the non-polar 32CA reactions have been classified into *pseudodiradical* type (*pdr-type*), *pseudoradical* type (*pmr-type*), carbenoid type (*cb-type*) and zwiterionic type (*zw-type*) in such way that the reactivity decreases in the order *pdr-type* < *pmr-type* = *cb-type* < *zw-type*.^20*b*^ The simplest azide, hydrazoic acid HN_3_, has a zwitterionic structure, thus participating in *zw-type* 32CA reactions. Although the 32CA reaction of hydrazoic acid with ethylene has a very high activation energy, 21.3 kcal mol^−1^, *zw-type* 32CA reactions can be accelerated by increasing the nucleophilic character of the TAC and the electrophilic character of the ethylene derivative, or *vice versa*.^21^

We herein provide a MEDT study, using DFT methods, that elucidates the mechanism of copper(i)-catalyzed azide–alkyne *zw-type* 32CA reaction, explaining the role of the catalyst in the different steps of the mechanism and its effect on the regioselective formation of 1,4-disubstitued 1,2,3-triazoles.

### Computational details

In the context of this study, all the possible reaction mechanisms, including intermediates and transition states, have been modeled and discussed in terms of relative energies obtained from quantum-mechanical calculations. The DFT method employing the B3LYP functional^22^ with the 6-31G(d) basis set^23^ has been used to carry out the full optimization of the compounds of interest in the gas phase with the G09 package.^24^ For Cu(i), LANL2DZ effective core potential has been used. It is stated that this methodology gives successful results for Cu metal and the DFT methodology with the B3LYP functional has been shown to give reliable results in transition metals, including Cu-catalyzed chemical reactions.^25–28^ The stationary points were analyzed by vibrational frequency calculations. All transition states were verified to be saddle points by one imaginary frequency belonging to the reaction coordinate. For all transition state structures the intrinsic reaction coordinate (IRC) was followed to validate the expected reactants and products.^29^ Solvation energies in water were added as single-point calculations using the conductor-like polarizable continuum model (CPCM). For comparative purposes, the azide–alkyne cycloaddition without Cu(i) catalysis have also been modeled.

The global electron density transfer (GEDT)^30^ was computed by the sum of the natural atomic charges (*q*), obtained by a natural population analysis (NPA),^31^ of the atoms belonging to each framework (f) at the TSs; GEDT = Σ*q*_f_. The sign indicates the direction of the electron density flux in such a manner that positive values mean a flux from the considered framework to the other one.

### Theoretical background

From a theoretical point of view, the electrophilic and nucleophilic behaviors of organic molecules can be characterized by using the reactivity indices defined within the conceptual DFT (CDFT) framework.^32^ Thus, Parr^33^ introduced the following definition of the electrophilicity index *ω* as:*ω* = *μ*^2^/2*η*, *μ*= (*E*_H_ + *E*_L_)/2 and *η*= (*E*_L_ − *E*_H_)where *μ* is the chemical potential and *η* is the absolute hardness.^34^ The electrophilicity index *ω* is a measure of the energy stabilization of a given molecule when it gains an amount of electron density.

Since the electron density donation process from a neutral molecule is thermodynamically unfavorable; we can assert that the best nucleophiles are those having low ionization potentials. Based on this idea, Domingo introduced an empirical (relative) nucleophilicity index^35^ (*N*) based on the HOMO energies obtained within the Kohn–Sham scheme,^36^ defined as:*N* = *E*_HOMO_ (Nu) − *E*_HOMO_ (TCE)

Nucleophilicity is referred to tetracyanoethylene (TCE) because it presents the lowest HOMO energy in a large series of molecules already investigated in the context of polar cycloadditions. This choice allows us to conveniently handle a nucleophilicity scale of positive values. Based on electron localization function bonding analysis along the reaction paths associated with C–C bond formation processes in polar reactions, Domingo has recently reported a new local reactivity index, named the local electrophilic, *P*_k_^+^, and nucleophilic, *P*_k_^−^, Parr functions which are obtained from the analysis of the Mulliken atomic spin density (ASD) at the radical anion and at the radical cation of the corresponding reagents.^37^ Such indexes are given by the following equations:*P*^−^(*r*) = *ρ*_s_^rc^(*r*) for electrophilic attacks*P*^+^(*r*) = *ρ*_s_^ra^(*r*) for electrophilic attackswhere *ρ*_s_^rc^(*r*) is the ASD of the radical cation, and *ρ*_s_^ra^(*r*) is the ASD of the radical anion. Each ASD condensed at the different atoms of the radical cation and radical anion provides our local nucleophilic *P*_k_^−^ and electrophilic *P*_k_^+^ Parr functions of the neutral system. With these electrophilic and nucleophilic Parr functions in hand, the local electrophilicity *ω*_k_ and the local nucleophilicity *N*_k_ indices can be redefined as follows:^37^*ω*_k_ = *ωP*_k_^+^*N*_k_ = *NP*_k_^−^

Therefore, one can easily find the *ω*_max_ and *N*_max_, which are associated with the most electrophilic and most nucleophilic centers in a molecule, respectively, and correspond to the centers with the highest electron density developed along the GEDT process.^30^

## Results and discussion

### Global and local electrophilicity/nucleophilicity index analysis

The 32CA reactions under study have been analyzed using global indexes defined in the context of CDFT.^32^ Studies devoted to the Diels–Alder and 32CA reactions have shown that the global indexes are powerful tools that enable understanding the behavior of polar cycloadditions.^38,39^Table 1 shows the static global properties, namely, electronic chemical potential *μ*, chemical hardness *η*, global electrophilicity *ω*, and global nucleophilicity *N* for the methylazide, methylacetylene and for the two complexes, namely dinuclear Cu(i)-acetylide, and reactive complex.

**Table 1:** Electronic chemical potential (*μ*, in eV), chemical hardness (*η*, in eV), global electrophilicity (*ω*, in eV) and global nucleophilicity *N* of methylazide, methylacetylene, dinuclear Cu(i)-acetylide and reactive complex

	*μ*	*η*	*ω*	*N*	
Methylazide	−3.85	6.18	1.19	2.17	
Methylacetylene	−2.68	8.74	0.41	2.06	
Dinuclear Cu(i)-acetylide	−1.78	0.91	1.74	5.88	
Reactive complex	−3.02	2.11	2.15	4.05	

From Table 1, we can notice that the electronic chemical potential of the dinuclear Cu(i)-acetylide, *μ* = −1.78 eV, is higher than that of methylazide, *μ* = −3.85 eV, indicating that at the TSs, the GEDT^30^ will takes place from the dinuclear Cu(i)-acetylide fragments towards the methylazide one in clear complete agreement with the GEDT computed at the corresponding TSs (*vide infra*).

Methylazide is a moderate electrophile, *ω* = 1.19 eV, and a moderate nucleophile, *N* = 2.17 eV within the electrophilicity^38^ and nucleophilicity^40^ scales. On the other hand, methylacetylene has an electrophilicity *ω* index of 0.41 eV, and a nucleophilicity *N* index of 2.06 eV, being classified as a marginal electrophile and a moderate nucleophile. The low electrophilic and nucleophilic character of methylazide and methylacethylene indicate that the corresponding 32CA reaction will have a low polar character. This is confirmed by the computed GEDT at the corresponding TSs (*vide infra*).

Coordination of the copper(i) to the carbon (C5) atom of methylacetylene increases the electrophilicity *ω* index of the corresponding complex dinuclear copper(i)-acetylide complex to 1.74 eV, but more markedly its nucleophilcity *N* index to 5.88 eV. This high value indicates that this Cu(i) complex will participate as a strong nucleophile in 32CA reactions with a large polar character.

Along a polar reaction, the bond breaking and bond forming processes take place at a specific position of a molecule, and if a molecule has several positions with similar reactivity; we should address the regio- or chemoselectivity issues of the reaction. This situation is common in cycloaddition reactions, in which the different approach modes of a reagent towards the other can yield two competitive isomers named regioisomers.

Recent studies focused on polar cycloaddition reactions have shown that the most favorable regioisomeric channel is that involving the bond formation between the most electrophilic and the most nucleophilic center of the reagents.^30^ Consequently, it is desirable to have local reactivity indices able to characterize these relevant centers in organic molecules.^37^ Cycloaddition reactions with a large polar character have shown that the analysis of the local electrophilicity *ω*_k_ at the electrophilic reagent and the local nucleophilicity *N*_k_ at the nucleophilic one derived from Parr functions allows explaining the regioselectivity that is experimentally observed. So, the values of the electrophilic and nucleophilic Parr functions, the local electrophilicity and the local nucleophilicity at the methylazide, dinuclear copper–acetylide complex and reactive complex are calculated and summarized in Table 2.

**Table 2:** Electrophilic and nucleophilic Parr functions, local electrophilicity, *ω*_k_, and nucleophilicity, *N*_k_, values (in eV)

	Number of atom	*P*_k_^+^	*P*_k_^−^	*ω*_k_	*N*_k_
Methylazide	N1	−0.01	**0.65**	−0.01	**1.40**
	N3	0.56	0.45	0.67	0.97
Dinuclear Cu(i)-acetylide	C4	−0.04	−0.06	−0.06	−0.37
	C5	−0.01	0.16	−0.01	0.96
	Cu6	**0.43**	0.16	**0.75**	0.94
	Cu7	0.41	0.16	0.71	0.93
Reactive complex	N1	0.05	0.09	0.10	0.36
	N3	**0.42**	0.07	**0.91**	0.26
	C4	−0.01	**0.26**	−0.02	**1.06**
	C5	0.01	0.12	0.02	0.49

**Fig. 3:**
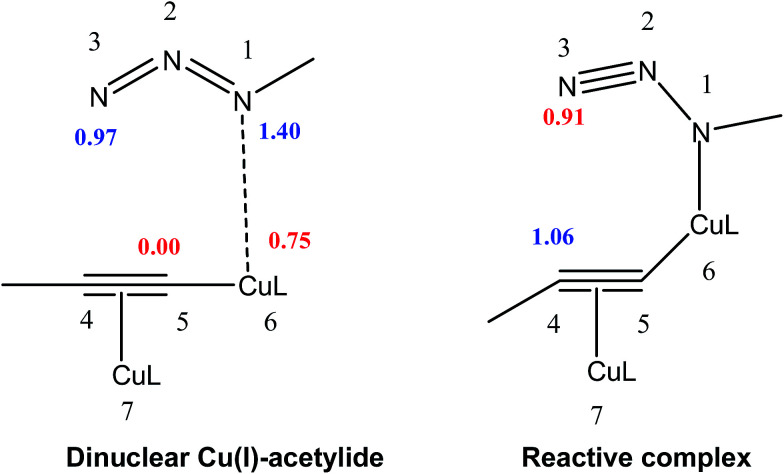
Local nucleophilicites *N*_k_, (in eV in blue) and local electrophilicites *ω*_k_ (in eV in red) calculated using Parr function.

Analysis of the local electrophilicity *ω*_k_ at dinuclear copper(i)-acetylide complex indicates that the Cu6 copper is the more electrophilically activated center of this intermediate, *ω*_Cu6_ = 0.75 eV, and analysis of the local nucleophilicity at organoazide indicates that the N1 nitrogen is the most nucleophilic center, *N*_N1_ = 1.4 eV. Consequently, along a polar process, the most favorable single bond formation will correspond to the N1–Cu6, leading to reactive complex, which has the largest electrophilic activation at the N3 nitrogen, *ω*_N3_ = 0.91 eV, and the largest nucleophilic activation at C4 carbon, *N*_C4_ = 1.06 eV (see Table 2, Fig. 3). In its intramolecular mode, both nucleophilic and electrophilic frameworks are present in the same molecule. Consequently, formation of the first C–N single bond will take place through the nucleophilic attack of the C4 carbon of the acetylide on the N3 of the azide, in complete agreement with the regioselectivity experimentally observed. Such an electron density transfer fact, in a donor–acceptor manner, from C4 to N3 has also been observed recently by Tüzün^17^ using Natural Bond Orbital (NBO) analyses to explain the formation of *σ*_C4–N3_ in the intermediate complex (IC) (see Table 3) but contradicts the one found by Quirante using QTAIM results, while explaining the electronic nature of the mechansim.^18^ The regioselective formation of 1,4 *versus* 1,5-disubstitued 1,2,3-triazole was also confirmed by using the Local Fukui Function and Dual Descriptor as different CDFT methodologies to the one thoroughly used in this study (Table S1 in ESI material†).

**Table 3:** Optimized geometries of the stationary points involved in the Cu(i)-catalyzed 32CA reaction. The lengths are given in angstroms, while the angles are given in degrees

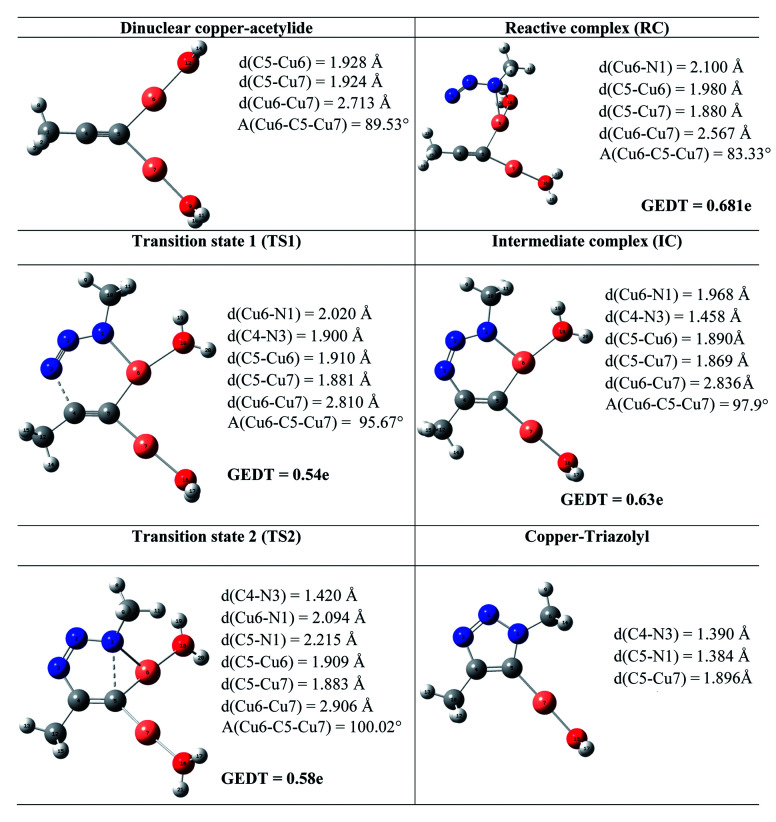

### Uncatalyzed azide–alkyne 32CA reaction

First, the uncatalyzed 32CA reaction of organic azides with alkynes was studied by means of DFT B3LYP/6-31G(d) calculations. This study showed that this 32CA reaction presents a high energy barriers for both the 1,4- and 1,5-approach modes. The energy barriers for the coupling of methylazide and propyne were computed in order to properly compare their energetics with those of the copper(i) catalyzed pathways described through this study.

**Fig. 4:**
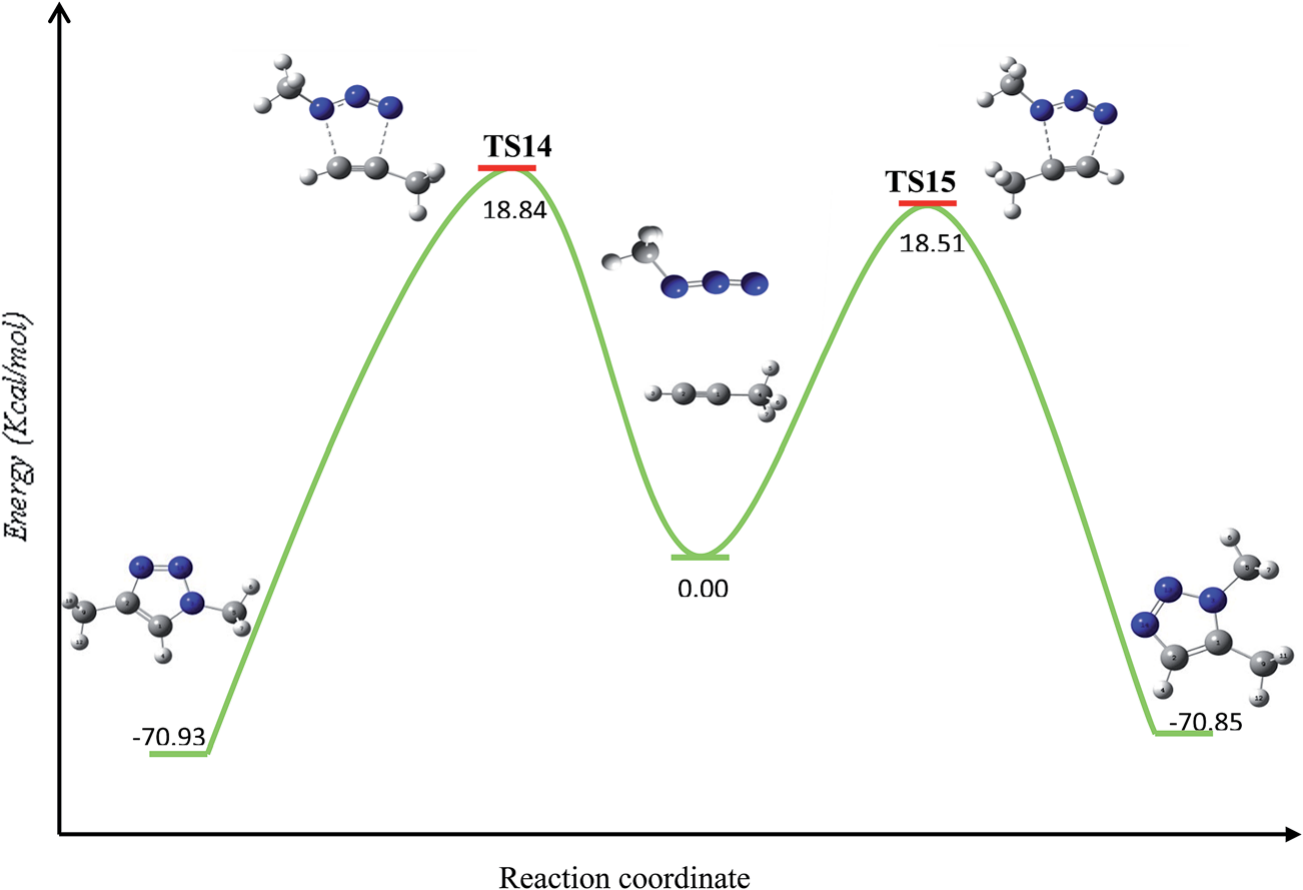
Calculated energy (kcal mol^−1^) barriers for the uncatalyzed thermal azide–alkyne 32CA reaction in absence of copper(i) species.

Our calculations provide, as expected, analogous energy barriers for the 1,4- and 1,5-regiochemistries (Fig. 4), resulting in 18.84 (TS14) and 18.51 (TS15) kcal mol^−1^, respectively. The corresponding energy difference, 0.27 kcal mol^−1^, explains the lack of regioselectivity when the 32CA reaction is carried out in the absence of any catalyst as well as the slowness of the transformation. The formation of triazoles is highly exothermic by 70.85 kcal mol^−1^ and 70.93 kcal mol^−1^, respectively.

B3LYP/6-311G(d,p) single point energy calculations at the stationary points involved in the 32CA reaction of methylazide with methylacetylene were performed (see Table S2 in ESI material†). The activation energies increase by 2.2 and 2.6 kcal mol^−1^, and the exothermic character of the reaction decreases by 7.5 as a consequence of a higher stabilization of methylazide that the other stationary point. Non substantial changes are found with the B3LYP/6-31G(d) analysis.

**Fig. 5:**
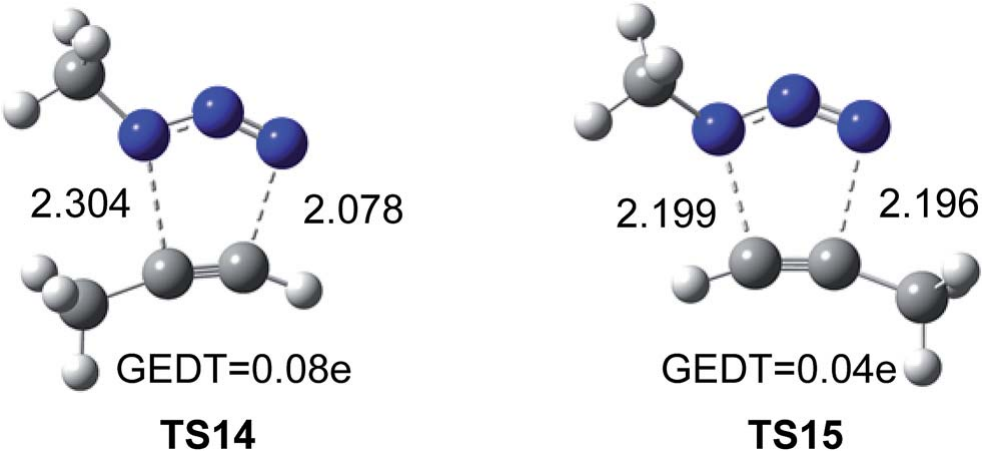
Optimized geometries of the regioisomeric TSs, TS14 and TS15, associated with the uncatalyzed 32CA reaction of methyl azide and propyne. The distances are given in Å.

The geometries of the TSs associated to the 32CA reactions between methyl azide and methylacetylene are given in Fig. 5. The lengths of the C–N forming bonds at the regioisomeric TSs are: 2.196 (C4–N3) and 2.199 (C5–N1) Å at TS14, and 2.304 (C4–N1) and 2.078 (C5–N3) Å at TS15. The extent of the asynchronicity of the bond formation in a 32CA reactions can be measured through the difference between the lengths of the two single bonds that are being formed in the reaction, Δ*r* = dist1 − dist2 (in Å). The asynchronicity at the TSs is 0.22 at TS14 and 0.01 at TS15. These results indicate that the 1.5-regioisomer process is more asynchronous than the 1.4-regioisomer one.

Numerous studies have shown a strong relationship between the polar character and the feasibility of organic reactions;^30^ the larger the GEDT at the TS is, the more polar and thus the faster is the reaction. In order to evaluate the electronic nature, *i.e.* polar or non-polar of the 32CA reaction between methylazide and propyne, the GEDT at the TSs was analysed. The resulting values are reported in Fig. 5. The natural charges at the TSs appear to be shared between the methylacetylene and methylazide. The GEDT, which fluxes from alkyne to azide at the TSs, is 0.04e at TS14 and 0.08*e* at TS15. These very low values indicate that these TSs have a non-polar character, in agreement with the low electrophilic character of methylazide and the low nucleophilic character of methylacethylene.

### Cu(i)-catalyzed stepwise mechanism

**Fig. 6:**
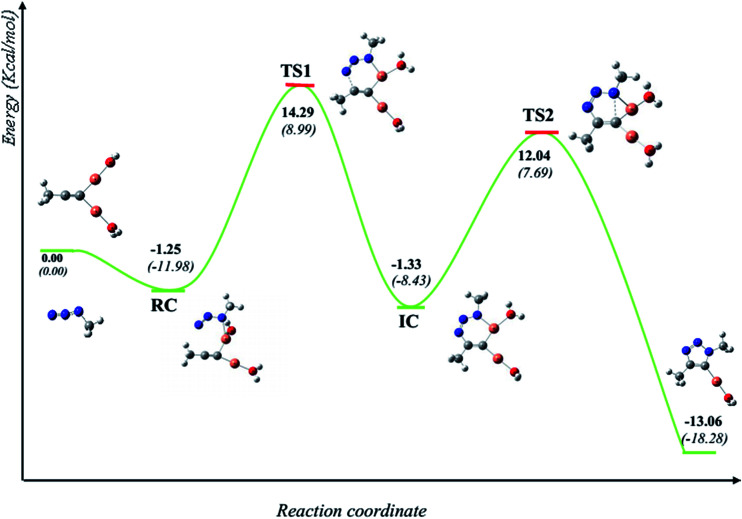
Schematic representation (energy (kcal mol^−1^) *vs.* reaction coordinate) of the reaction of copper(i)-catalyzed 32CA between methyl azide and propyne (bold numbers for gas phase and numbers between brackets for water as reaction medium).

It is generally accepted that the active catalyst comprises copper in the oxidation Cu(i). The alkyne substrates bind to copper(i) in a π-coordination mode, in such a way the acidity of the terminal alkyne proton increases significantly due to the formation of stable μ-acetylide copper(i) intermediates.^41^ Kinetic measurements have shown that the rate of the ligand-free CuAAC reaction is second order, depending on the concentration of copper(i) ions present in the reaction mixture.^42^ These findings have led to a mechanistic proposal for the CuAAC based on quantum-mechanical model calculations.^16^ In the first step of this mechanism, a terminal alkyne binds to a copper(i) center as a π-ligand. This coordination significantly increases the acidity of the alkyne terminal proton because a stable dinuclear Cu(i)-acetylide complex can be formed upon deprotonation. The organoazide can bind reversibly to the copper atom *via* the nitrogen proximal to carbon, forming a reactive complex RC. This is effectively a starting point for the stepwise sequence represented in Fig. 6. This step is slightly exothermic computationally by 1.25 kcal mol^−1^ (11.98 kcal mol^−1^ in water). After that, the distal nitrogen of the azide in RC can bind to the C-2 carbon of the acetylide, forming the intermediate reactional complex IC. The calculated barrier is 14.29 kcal mol^−1^ (8.99 kcal mol^−1^ in water), which is considerably lower than the barrier for the uncatalyzed reaction (18.51 and 18.84 kcal mol^−1^). The subsequent N–C single bond formation is usually considered as the rate-limiting step at least for standard CuAAC catalysts. This explains the enormous rate acceleration of the Cu(i)-catalyzed process, 7 to 8 orders of magnitude, as compared to the purely thermal cycloaddition process. The stability of the intermediate six-membered cupracycle determines the energy of the TS in the present mechanistic model, which takes into account tow copper centers, a strain less and thus quite stable cyclic intermediate with sp^2^ hybridized carbon atom is formed. With two attached copper atoms, an sp^2^ hybridized carbon atom does not lead to any ring strain. In the next step, a triazolide ligand is formed by reductive elimination. From this intermediate, the barrier for ring contraction, which forms the triazolyl-copper derivative is 13.37 kcal mol^−1^ (16.12 kcal mol^−1^ in water). TS2 is 2.25 kcal mol^−1^ (1.3 kcal mol^−1^ in water) lower than TS1, which is similar to the very recent computational results that proved the binuclear nature of the CuAAC mechanism by involving ligand exchanges on the copper centers.^17^ The optimized TSs and intermediates are shown in Table 3. The last step corresponds to a fast protonation of the copper triazolide, leading to the release of the triazole product; meanwhile an active copper species catalyst is regenerated, thereby closing the catalytic cycle.

The reaction has also been considered in water as solvent. The relative energies are systematically lower than the gas phase results for starting reagents, reactive complex, intermediate complex and transition states (see Fig. 6). We noticed that inclusion of the water as a solvent has not changed the picture of the mechanism.

The geometries of the TSs and the intermediate involved in the Cu(i)-catalyzed 32CA reaction are given in Table 3. At the reactive complex associated with the nucleophilic attack of the azide by nitrogen atom N1 at the dinuclear Cu(i)-acetylide, the length of the Cu6–N1 forming bond is 2.100 Å. The C4–N3 bond length at the corresponding TS1 is 1.900 Å, while the distance between the Cu6 and N1 nitrogen atom becomes 2.020 Å. The Cu6–N1 and C4–N3 bonds length at the corresponding intermediate complex (IC) are 1.968 and 1.458 Å respectively. Finally, at TS2 associated with ring-closure process, the length of the C5–N1 forming bond is 2.215 Å.

The computed NPA atomic charges were partitioned between the organoazide and the acetylide frameworks. The corresponding GEDT values are reported in Table 3. The GEDT developed along the nucleophilic attack of the propyne framework to the azide one is: 0.68*e* at RC, 0.54*e* at TS1, 0.63*e* at IC and 0.58*e* at TS2. The high polar character of this *zw-type* 32CA reaction is in clear agreement with the large increase in the nucleophilicity of alkyne with the coordination to the copper(i).

## Conclusion

The mechanisms of the *zw-type* 32CA reactions of organoazides with alkynes in the absence and in the presence of a copper(i) catalyst have been studied within the MEDT using DFT methods at the B3LYP/6-31G(d) (LANL2DZ for Cu) computational level. For the uncatalysed 32CA reaction, two regioisomeric reactive channels were studied in the absence of a copper(i) catalyst, showing that the 32CA reaction takes place through an asynchronous one-step mechanism with a very non-polar character. The two regioisomeric reactive paths present similar high activation energies.

Coordination of copper(i) to alkyne produces relevant changes in this *zw-type* 32CA reaction as a consequence of the large enhancement in the nucleophilicity of the corresponding dinuclear Cu(i)-acetylide complex. Formation of the experimentally observed 1,4-tiazole takes place through a stepwise mechanism with formation of an intermediate complex. Analysis of the CDFT global and local electrophilicity/nucleophilicity indices allows explaining correctly the behaviors of the copper(i) catalyzed *zw-type* 32CA reaction. Coordination of the copper to alkyne changes the mechanism from a non-polar one-step mechanism to a polar stepwise one, as a consequence of the large nucleophilic character of the dinuclear Cu(i)-acetylide complex. Analysis of the local indexes allows characterizing the more nucleophilic/electrophilic center of the reagent. Regioselectivity is correctly explained by means of the favorable two-center interaction that takes place along the 1,4 reaction path.

## Conflicts of interest

There are no conflicts to declare.

## Supplementary Material

RA-008-C7RA10653J-s001
