# Supplementary material for: Understanding the mechanism and regioselectivity of the copper(i) catalyzed [3 + 2] cycloaddition reaction between azide and alkyne: a systematic DFT study
Source: RSC Adv. 2018 Feb 16;8(14):7670–8. doi: 10.1039/c7ra10653j (PMC9078510; doi:10.1039/c7ra10653j)
Supplement: RA-008-C7RA10653J-s001 [file RA-008-C7RA10653J-s001.pdf]

Electronic Supplementary Material (ESI) for RSC Advances.
This journal is © The Royal Society of Chemistry 2018

Revised Version

Supplementary Information

Understanding the mechanism and regioselectivity of the copper(I) catalyzed [2+3] cycloaddition reaction between azide and alkyne: A systematic DFT study†,‡

Hicham Ben El Ayouchia,[a] Lahoucine Bahsis,[a] Hafid Anane, [a,*] Luis R. Domingo,[b] and Salah-Eddine Stiriba[a,c,*]

[a]Laboratoire de Chimie Analytique et Moléculaire, - LCAM, Faculté Polydisciplinaire de Safi, Université Cadi Ayyad, Safi 46030, Morocco.
[b]Departamento de Química Orgánica, Universidad de Valencia, Avda. Dr. Moliner 50, 46100 Burjassot, Valencia, Spain.
[c]Instituto de Ciencia Molecular/ ICMol, Universidad de Valencia, C/Catedrático José Beltrán Nº 2, 46980 Paterna, Valencia, Spain.

Contents:

S1 Theoretical background.

S4 Table S1 with the electrophilic and nucleophilic Fukui functions, local Electrophilicity, ω_k, Nucleophilicity, N_k, and Dual Descriptor Values.

S4 Table S2 with the B3LYP/6-31G(d) and B3LYP/6-311G(d,p) total and relative energies of the stationary points involved in the 32CA reaction of methylazide with methylacetylene.

S5 Cartesian coordinates, energies, and imaginary frequency (transition states) for all optimized geometries.

Theoretical background

Along with the global indices, the conceptual DFT defines a number of local indices. In 1984, Parr and Yang [1] proposed within the DFT the f(r) function named frontier function or Fukui function for a molecule, which was defined as:

$$f_K = \left[\frac{\partial\rho(r)}{\partial N}\right]_{v(r)}$$

Parr and Yang assumed that the preferred direction for the approach of a reagent towards the other is the one for which the initial variation of the chemical potential μ for a species is a maximum, and that the preferred direction is the one with the largest f(r) at the reaction site [1]. Using a frozen core approximation, in which valence $\delta\rho = \delta\rho_{valence}$, they proposed:

$$f_k^- = \rho_{HOMO}(r) \qquad for\ electrophilic\ attacks$$

And

$$f_k^+ = \rho_{LUMO}(r) \qquad for\ nucleophilic\ attacks$$

Although, in principle, the electron density of a neutral or $N_{0\,\pm\,1electron}$ molecule contains all the information needed for the evaluation of the Fukui function f (r), most studies in the literature have been carried out in the so-called finite difference method, in which the Fukui functions f (r) are approximated as [2]:

$$f_k^- = [q_k(N) - q_k(N-1)] \quad for\ electrophilic\ attacks$$

And

$$f_k^+ = [q_k(N+1) - q_k(N)] \qquad for\ nucleophilic\ attacks$$

where $q_k(N)$, $q_k(N-1)$ and $q_k(N+1)$ are the atomic charges in the neutral, cationic and anionic species respectively.

Thus, the local electrophilicity [3] ω_k and the local nucleophilicity [4] N_k indices were defined as:

$$\omega_k = \omega f_k^+$$

$$N_k = N f_k^-$$

Analysis of the local electrophilicity ω_k and local nucleophilicity N_k indices in an organic molecule allows for the characterization ofthe most electrophilic and nucleophilic centers in the molecule, and thereby to predict theregio- and chemoselectivity in a polar reaction.

Morell et al.[5-7] have proposed a local reactivity descriptor (LRD) which is called the dual descriptor (DD) Δf(r). The dual descriptor can be condensed over the atomic sites: when $\Delta f_k > 0$ the process is driven by a nucleophilic attack on atom k and then that atom acts as an electrophilic species. Conversely, when $\Delta f_k < 0$ the process is driven by an electrophilic attack over atom k and therefore atom k acts as a nucleophilic species. Thus, the Dual Descriptor was defined as:

$$\Delta f_k(r) = f_k^+ - f_k^-$$

References

1 R.G. Parr, W. Yang; *J. Am. Chem. Soc.* **1984**, *106*, 4049.

2 W. Yang, W.J. Mortier; *J. Am. Chem. Soc.* **1986**, *108*, 5708.

3 L.R. Domingo, M.J. Aurell, P. Perez, R. Contreras; J. *Phys. Chem. A*, **2002**, 106, 6871.

4 P. Pérez, L. R. Domingo, M. Duque-Noreña, E. Chamorro; *J. Mol. Struct. (Theochem)* **2009**, *895*, 86.

5 C. Morell, A., Grand, A. Toro-Labbé,. *J. Phys. Chem. A*, **2005**, *109*, 205.

6 C. Morell, A. Hocquet, A., Grand and B, Jamart-Gregoire, *J. Mol. Struct. (Theochem)*, **2008**, *849*, 46

7 C. Morell, P. Ayers, A. Grand, S. Gutiérrez-Oliva, and A. Toro-Labbé, *Phys. Chem. Chem. Phys.*, **2008**, *10*, 7239.

Table S1. Electrophilic and nucleophilic Fukui functions, local Electrophilicity, ω_k, Nucleophilicity, N_k, and Dual Descriptor Values (in eV).

	Number of atom	f_k^+	f_k^-	ω_k	N_k	$\Delta f_k(r)$
Methylazide	N1	0.18	**0.43**	0.22	**0.93**	**-0.24**
	N3	0.40	0.40	0.47	0.88	0.01
Dinuclear Cu(I)-acetylide	C4	0.05	0.08	0.09	0.48	-0.03
	C5	0.01	-0.03	0.02	-	0.04
	Cu6	**0.37**	0.30	**0.66**	0.52	**0.07**
	Cu7	0.37	0.29	0.66	0.50	**0.08**
Reactive complex	N1	0.01	0.13	0.02	0.55	-0.12
	N3	**0.13**	0.28	0.28	**1.17**	**-0.15**
	C4	0.03	**0.02**	0.07	0.11	0.01
	C5	-0.02	0.00	-	0.01	-0.02

Table S2. B3LYP/6-31G(d) and B3LYP/6-311G(d,p) total (E, in a.u.) and relative energies (ΔE, kcal mol^{-1}) of the stationary points involved in the 32CA reaction of methylazide with methylacetylene.

	B3LYP/6-31G(d)		B3LYP/6-311G(d,p)	
	E	ΔE	E	ΔE
Methylazide	-204.093315		-204.148221	
Methylacetylene	-116.653270		-116.690652	
**TS14**	-320.716560	18.84	-320.805340	21.04
**TS15**	-320.717077	18.52	-320.805182	21.14
**CA14**	-320.859635	-70.95	-320.940029	-63.48
**CA15**	-320.859495	-70.86	-320.939704	-63.28

Cartesian coordinates, energies, and imaginary frequency (transition states) for all optimized geometries.

Methyl azide

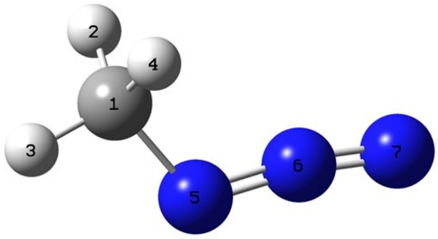

E(Hartree) = -204.093315062

Standard orientation:

Center Number	Atomic Number	Atomic Type	Coordinates (Angstroms) X	Y	Z
1	7	0	0.391276	-0.630665	-0.000048
2	7	0	-0.717975	-0.096025	0.000111
3	7	0	-1.801285	0.271185	-0.000057
4	6	0	1.554423	0.283851	-0.000006
5	1	0	1.563227	0.917762	-0.893423
6	1	0	1.564295	0.916251	0.894472
7	1	0	2.441839	-0.348587	-0.001054

Propine

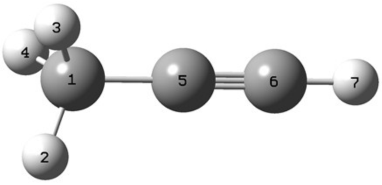

E(Hartree) = -116.65327005

Standard orientation:

Center Number	Atomic Number	Atomic Type	Coordinates (Angstroms) X	Y	Z
1	6	0	0.000000	0.000000	-1.241443
2	1	0	0.000000	1.022446	-1.638037
3	1	0	0.885464	-0.511223	-1.638037
4	1	0	-0.885464	-0.511223	-1.638037
5	6	0	0.000000	0.000000	0.218960

6	6	0	0.000000	0.000000	1.426135
7	1	0	0.000000	0.000000	2.492199

TS14

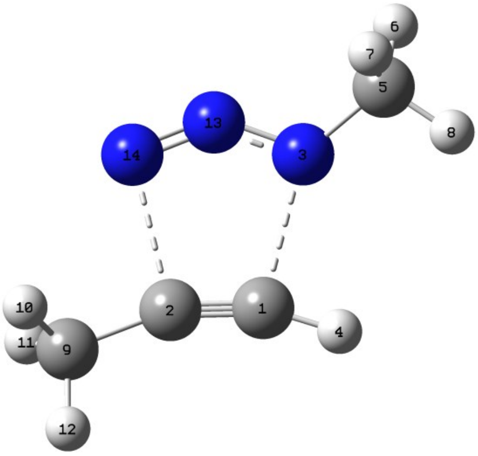

Imaginary Frequency: - 424.22 cm^{-1}

E(Hartree) = -320.71656009

Standard orientation:

Center Number	Atomic Number	Atomic Type	Coordinates (Angstroms) X	Y	Z
1	6	0	0.487394	1.337557	-0.100523
2	6	0	1.410783	0.522122	-0.003895
3	7	0	-1.333364	0.116647	-0.283477
4	1	0	-0.042987	2.258541	-0.198025
5	6	0	-2.690368	0.300780	0.223654
6	1	0	-3.412403	-0.325901	-0.313834
7	1	0	-2.771957	0.102121	1.300074
8	1	0	-2.948619	1.346396	0.039727
9	6	0	2.780003	-0.004344	0.125572
10	1	0	2.881184	-0.606649	1.034668
11	1	0	3.029571	-0.655126	-0.719285
12	1	0	3.513375	0.809409	0.164461
13	7	0	-0.749641	-0.985968	-0.095490
14	7	0	0.343713	-1.397177	-0.017695

CA14

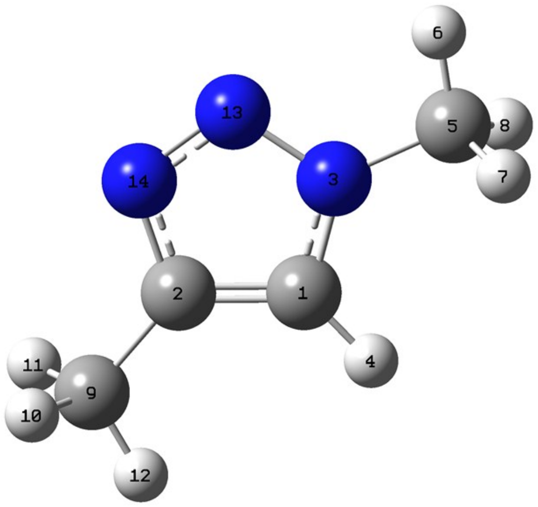

E(Hartree) = -320.85963450

Standard orientation:

Center Number	Atomic Number	Atomic Type	Coordinates (Angstroms)		
			X	Y	Z
1	6	0	-0.014549	-0.930555	-0.000091
2	6	0	-1.106754	-0.088294	-0.000003
3	7	0	1.061279	-0.100403	-0.000003
4	1	0	0.079239	-2.006253	-0.000119
5	6	0	2.476080	-0.421935	0.000080
6	1	0	3.018498	0.524292	-0.000062
7	1	0	2.742360	-0.995596	0.893316
8	1	0	2.742384	-0.995883	-0.892963
9	6	0	-2.568754	-0.407312	0.000099
10	1	0	-3.062708	0.014431	0.882680
11	1	0	-3.063038	0.015653	-0.881703
12	1	0	-2.735865	-1.488724	-0.000604
13	7	0	0.661995	1.190806	-0.000025
14	7	0	-0.642847	1.198261	-0.000122

TS15

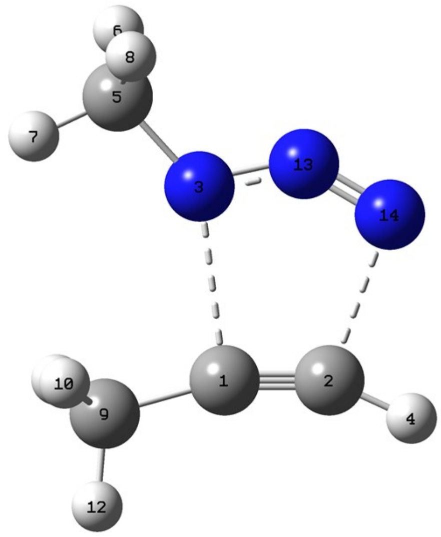

Imaginary Frequency: - 417.12 cm^{-1}

E(Hartree) = -320.71707686

Standard orientation:

Center Number	Atomic Number	Atomic Type	Coordinates (Angstroms)		
			X	Y	Z
1	6	0	1.424277	-0.069331	0.013263
2	6	0	1.457803	1.165713	0.039689
3	7	0	-0.866149	-0.301062	-0.089603
4	1	0	1.932082	2.122596	0.072124
5	6	0	-1.962074	-1.253320	0.052822
6	1	0	-2.700235	-1.160260	-0.754003
7	1	0	-1.517765	-2.249923	-0.008126
8	1	0	-2.475560	-1.163434	1.018954
9	6	0	1.779587	-1.492503	-0.014312
10	1	0	1.364064	-2.026496	0.847059
11	1	0	1.406394	-1.978233	-0.922037
12	1	0	2.870362	-1.610167	0.009258
13	7	0	-1.099202	0.935100	-0.030360
14	7	0	-0.474206	1.932042	0.003963

CA15

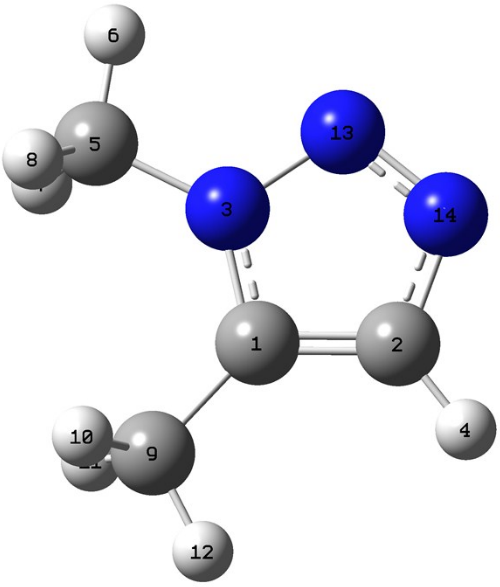

E(Hartree) = - 320.85949506

Standard orientation:

Center Number	Atomic Number	Atomic Type	Coordinates (Angstroms) X	Y	Z
1	6	0	0.307114	-0.694774	0.000023
2	6	0	-0.972146	-1.212975	-0.000003
3	7	0	0.098191	0.652085	0.000005
4	1	0	-1.284711	-2.247865	-0.000084
5	6	0	1.068169	1.729722	0.000001
6	1	0	0.506701	2.664573	-0.000105
7	1	0	1.701127	1.684421	0.892330
8	1	0	1.701292	1.684308	-0.892207
9	6	0	1.657420	-1.332011	0.000094
10	1	0	2.243802	-1.052109	-0.884381
11	1	0	2.243376	-1.052797	0.885069
12	1	0	1.555056	-2.420414	-0.000350
13	7	0	-1.228877	0.940396	0.000232
14	7	0	-1.873597	-0.192466	-0.000374

dinuclear copper-acetylide

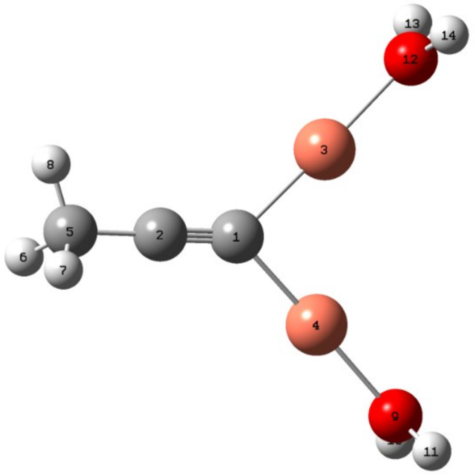

E(Hartree) = -661.22746111

Standard orientation:

Center Number	Atomic Number	Atomic Type	Coordinates (Angstroms) X	Y	Z
1	6	0	-0.069065	1.025164	0.000026
2	6	0	-0.185563	2.272173	0.000047
3	29	0	-1.318768	-0.444032	0.000005
4	29	0	1.386982	-0.233681	-0.000004
5	6	0	-0.333717	3.734833	-0.000025
6	1	0	0.126867	4.181930	0.891087
7	1	0	0.134490	4.182558	-0.886854
8	1	0	-1.395200	4.021644	-0.004532
9	8	0	2.883855	-1.478851	-0.000007
10	1	0	3.361371	-1.758149	0.820447
11	1	0	3.361069	-1.758871	-0.820390
12	8	0	-2.623429	-1.889645	-0.000012
13	1	0	-3.060115	-2.230073	0.820602
14	1	0	-3.060028	-2.230406	-0.820533

RC

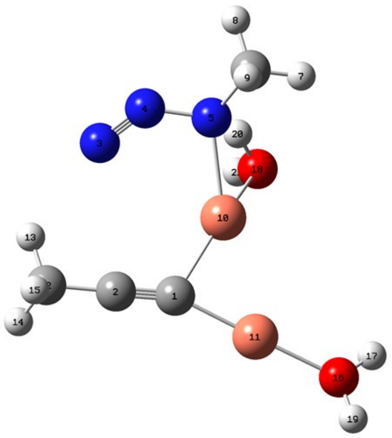

E(Hartree) = -865.32282171

Standard orientation:

Center Number	Atomic Number	Atomic Type	Coordinates (Angstroms) X	Y	Z
1	6	0	0.764755	1.244781	-0.081985
2	6	0	0.193218	2.350187	-0.011102
3	7	0	-2.740172	1.300248	0.399201
4	7	0	-3.037059	0.280218	-0.211059
5	7	0	-2.308869	-0.928468	-0.279854
6	6	0	-2.911687	-1.836405	-1.281618
7	1	0	-2.374091	-2.790754	-1.226857
8	1	0	-3.976465	-2.022887	-1.075058
9	1	0	-2.825810	-1.447570	-2.307994
10	29	0	-0.308242	-0.410633	0.093427
11	29	0	2.205964	0.040099	-0.170991
12	6	0	-0.505609	3.638070	0.087429
13	1	0	-1.536522	3.449883	0.410688
14	1	0	-0.021919	4.300350	0.817540
15	1	0	-0.538306	4.151827	-0.882072
16	8	0	3.672436	-1.246032	-0.211450
17	1	0	3.546464	-2.159890	0.101495
18	8	0	-0.896115	-1.903103	1.661060
19	1	0	4.566139	-1.083600	-0.560903
20	1	0	-1.743806	-1.674670	1.123314
21	1	0	-0.981554	-1.727895	2.617966

TS1

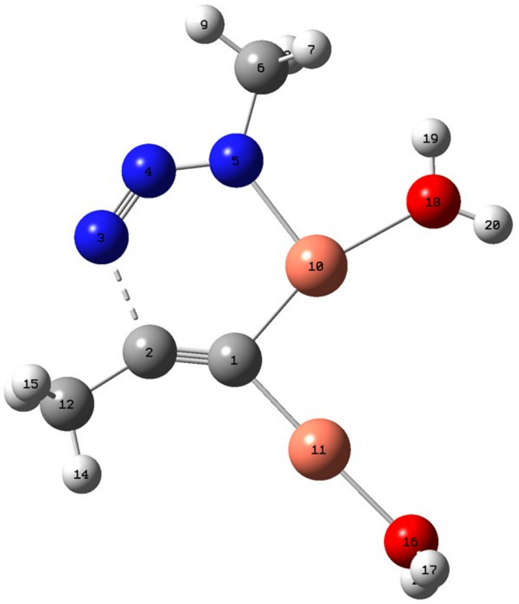

Imaginary Frequency: -328.76 cm^{-1}

E(Hartree) = -865.29799698

Standard orientation:

Center Number	Atomic Number	Atomic Type	Coordinates (Angstroms) X	Y	Z
1	6	0	0.465139	0.807646	0.000768
2	6	0	-0.035620	2.010202	0.001664
3	7	0	-1.927311	2.187567	-0.005040
4	7	0	-2.679646	1.192302	-0.005392
5	7	0	-2.436584	-0.132038	-0.013140
6	6	0	-3.681067	-0.933985	0.014166
7	1	0	-3.675930	-1.677166	-0.801437
8	1	0	-3.798135	-1.461757	0.976154
9	1	0	-4.559030	-0.291314	-0.125902
10	29	0	-0.536884	-0.818649	-0.002866
11	29	0	2.156612	-0.016127	0.001049
12	6	0	0.348321	3.458748	0.005487
13	1	0	-0.067255	3.961087	0.887926
14	1	0	1.439844	3.567105	0.008584
15	1	0	-0.062608	3.964285	-0.877292
16	8	0	3.917162	-0.929316	0.000293
17	1	0	4.449381	-0.998712	-0.817263
18	8	0	-0.944320	-2.823817	0.001334
19	1	0	-1.884458	-3.080462	-0.006972
20	1	0	-0.341757	-3.587294	0.021200
21	1	0	4.469256	-0.952676	0.807166

IC

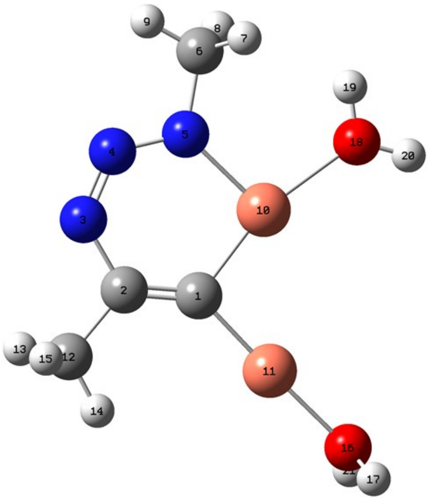

E(Hartree) = -865.32290919

Standard orientation:

Center Number	Atomic Number	Atomic Type	Coordinates (Angstroms) X	Y	Z
1	6	0	0.422049	0.723670	-0.000137
2	6	0	-0.129030	1.961247	-0.000106
3	7	0	-1.554373	2.271175	-0.000019
4	7	0	-2.514614	1.401985	0.000063
5	7	0	-2.357570	0.060941	0.000109
6	6	0	-3.661973	-0.640375	0.000185
7	1	0	-3.769168	-1.271791	-0.897818
8	1	0	-3.769542	-1.270926	0.898745
9	1	0	-4.476214	0.093903	-0.000339
10	29	0	-0.616154	-0.856252	-0.000068
11	29	0	2.111101	-0.078453	-0.000046
12	6	0	0.648557	3.270474	-0.000028
13	1	0	0.383253	3.864888	0.884756
14	1	0	1.727359	3.082556	-0.001102
15	1	0	0.381590	3.865967	-0.883568
16	8	0	3.869700	-0.992899	0.000258
17	1	0	4.404160	-1.077965	-0.811872
18	8	0	-1.187443	-2.819743	0.000036
19	1	0	-2.144417	-3.004851	0.000024
20	1	0	-0.644535	-3.626862	-0.000900
21	1	0	4.404287	-1.076135	0.812497

TS2

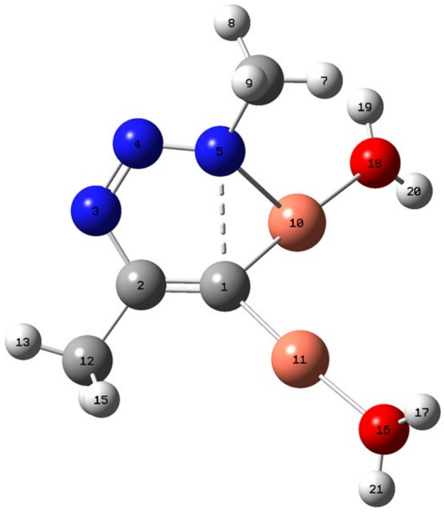

Imaginary Frequency: -185.73 cm^{-1}

E(Hartree) = -865. 30153680

Standard orientation:

Center Number	Atomic Number	Atomic Type	Coordinates (Angstroms) X	Y	Z
1	6	0	-0.226614	0.641091	-0.160092
2	6	0	0.241945	1.906241	-0.449060
3	7	0	1.552702	2.331657	-0.104519
4	7	0	2.248673	1.491183	0.635785
5	7	0	1.715712	0.263729	0.836097
6	6	0	2.323334	-0.438997	1.996316
7	1	0	2.078551	-1.507388	1.941204
8	1	0	3.414863	-0.310443	1.993362
9	1	0	1.934398	-0.044561	2.945079
10	29	0	0.748910	-0.948293	-0.572204
11	29	0	-1.918636	-0.099093	0.209316
12	6	0	-0.604945	3.012634	-1.055034
13	1	0	-0.045907	3.953876	-1.015081
14	1	0	-0.856175	2.797765	-2.102206
15	1	0	-1.548579	3.124320	-0.504094
16	8	0	-3.634804	-0.966587	0.664379
17	1	0	-3.812116	-1.469084	1.486575
18	8	0	1.668544	-2.637571	-1.081212
19	1	0	2.633843	-2.744202	-0.974059
20	1	0	1.285816	-3.275273	-1.711258
21	1	0	-4.454472	-0.649330	0.234575

Copper-Triazolyl

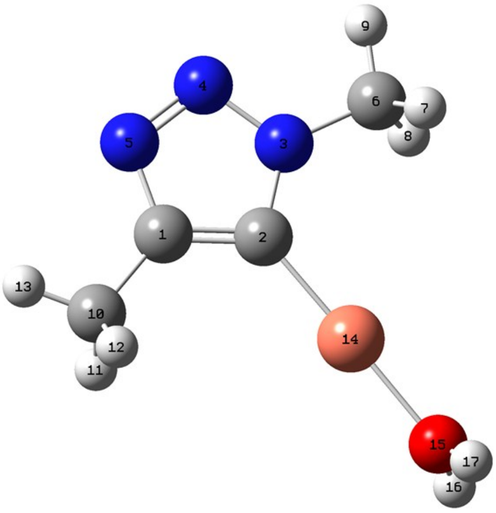

E(Hartree) = -592.79161005

Standard orientation:

Center Number	Atomic Number	Atomic Type	Coordinates (Angstroms)		
			X	Y	Z
1	6	0	-1.395398	1.098493	-0.001128
2	6	0	-0.471649	0.023276	0.000336
3	7	0	-1.305206	-1.082751	0.001274
4	7	0	-2.659573	-0.739149	0.000037
5	7	0	-2.696583	0.608640	-0.001486
6	6	0	-0.940256	-2.502068	0.000496
7	1	0	-0.361768	-2.749002	-0.895572
8	1	0	-0.344316	-2.744773	0.886101
9	1	0	-1.859093	-3.093136	0.010740
10	6	0	-1.124980	2.579180	0.000269
11	1	0	-0.588928	2.893127	0.905892
12	1	0	-0.513309	2.879435	-0.860532
13	1	0	-2.068283	3.135642	-0.043278
14	29	0	1.424713	0.017351	0.000160
15	8	0	3.355004	0.045967	-0.000618
16	1	0	3.901213	0.068042	0.806263
17	1	0	3.901014	0.039278	-0.807912

Water

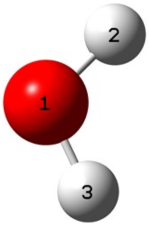

E(Hartree) = -76.40801899

Standard orientation:

Center Number	Atomic Number	Atomic Type	Coordinates (Angstroms) X	Y	Z
1	8	0	-0.791897	0.432781	0.000000
2	1	0	0.168103	0.432781	0.000000
3	1	0	-1.112351	1.337717	0.000000

Copper-Water

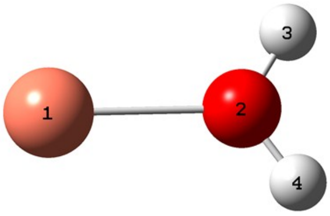

E(Hartree) = - 272.54997945

Standard orientation:

Center Number	Atomic Number	Atomic Type	Coordinates (Angstroms) X	Y	Z
1	29	0	0	0	0.556252
2	8	0	0	0	-1.50408
3	1	0	0	0.81386	-2.04933
4	1	0	0	-0.81386	-2.04933
